# The association between dietary approaches to stop hypertension diet and mediterranean diet with metabolic syndrome in a large sample of Iranian adults: YaHS and TAMYZ Studies

**DOI:** 10.1002/fsn3.2387

**Published:** 2021-06-11

**Authors:** Shirin Hassani Zadeh, Amin Salehi‐Abargouei, Masoud Mirzaei, Azadeh Nadjarzadeh, Mahdieh Hosseinzadeh

**Affiliations:** ^1^ Nutrition and Food Security Research Center Shahid Sadoughi University of Medical Sciences Yazd Iran; ^2^ Department of Nutrition School of Public Health Shahid Sadoughi University of Medical Sciences Yazd Iran; ^3^ Yazd Cardiovascular Research Centre Shahid Sadoughi University of Medical Sciences Yazd Iran

**Keywords:** dietary approaches to stop hypertension, mediterranean dietary pattern, metabolic syndrome

## Abstract

Dietary patterns are considered as a modifiable risk factor for metabolic syndrome (MetS). Therefore, the present study aimed to evaluate the association between the Dietary Approaches to Stop Hypertension (DASH) and Mediterranean (MED) dietary patterns and MetS. This cross‐sectional study was conducted on the data from recruitment phase of prospective studies on Iranian adults known as Yazd Health Study (YaHS) and Yazd Nutrition Study (TAMYZ). MetS was diagnosed among 2,221 adults based on the Adult Treatment Panel III criteria. The participants' dietary intake was assessed by a validated food frequency questionnaire. According to the predefined methods, DASH and MED scores were calculated. Multivariate logistic regression was used to evaluate the relationship of DASH and MED dietary patterns with MetS. The prevalence of MetS was 28.8% in the present study. Women who were in the highest quintile of DASH in comparison with those who were in the first quintile tended to decrease the odds of MetS after adjusting for the potential confounders (OR: 0.50, CI: 0.27–0.95). There was a significant decreasing trend in the odds of MetS across increasing quintiles of the DASH in women (*p*‐trend = .006). Also, the highest adherence to DASH reduced the odds of abdominal obesity (OR: 0.34, CI: 0.15–0.77) in women. Although adherence to MED dietary pattern had no significant relationship with MetS, moderate adherence to this dietary pattern could decrease the odds of fasting blood glucose levels (OR: 0.57, CI: 0.33–0.97) and abdominal obesity (OR: 0.42, CI: 0.20–0.87) in women. We found evidence indicating a significant protective association between DASH and METs and its component in women. Hence, more prospective studies are needed to confirm our findings in other populations.

## Graphical abstract

Adherence to healthy dietary patterns like DASH and Mediterranean (MED) can play an important role in preventing some chronic diseases. In this article, DASH and MED dietary patterns were considered at the same time in a large sample of Iranian adults. In addition, secondary analysis has been done based on gender. We found evidence indicating a significant protective association between DASH and MetS and its component in women.

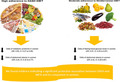

**Abbreviations**
BMI: body mass index
DASH: dietary approaches to stop hypertension
FBS: Fasting Blood Sugar
FCT: food composition table
FFQ: food frequency questionnaire
MED: mediterranean dietary pattern
MetS: metabolic Syndrome
OR: odds ratio
TAMYZ: TAghzieh Mardom‐e‐Yazd (Yazd Nutrition Study)
USDA: United States Department of Agriculture
YaHS: Yazd Health Study

## 1 INTRODUCTION

Metabolic syndrome (MetS) is a multifactorial disease with a collection of abnormalities such as abdominal obesity, dyslipidemia, high glucose levels, and high blood pressure (Linardakis et al., 2008). MetS increases the risk of cardiovascular disease, arthritis, chronic kidney disease, and type 2 diabetes (Lakka et al., 2002). In recent years, the prevalence of MetS has increased due to significant change in lifestyle and dietary habits (Lazo et al., 2013). A study conducted in Yazd, Iran, revealed that the prevalence of this disease is 33%, and it is more common in females (39.9%) than males (25.9%) (Mirzaei et al., 2017). A meta‐analysis study also confirmed this high prevalence of MetS in Iranian population especially in women (Fatahi et al., 2020). However, its prevalence is about 25% in the world (O'neill & O'driscoll, 2015). Recent findings represented that the individuals suffering this disease are 3–5 times more likely to get cardiovascular disease and die as a result, compared to nonaffected individuals (Salari et al., 2020). In addition, patients with MetS have a higher rate of hospitalization compared to patients without MetS (Alswat et al., 2017; Fitzmorris et al., 2015). Hence, identification of risk factors, especially modifiable risk factors like diet and lifestyle, can play a key role in the prevention of MetS (Reaven, 2005). Previous researches revealed that single dietary components, such as nutrients and food groups, could affect MetS. For example, a high intake of saturated fatty acids (Klemsdal et al., 2010), animal proteins (Lutsey et al., 2008), high GI food (LaHaye et al., 2005), and low intake of whole grains and fiber (Castanho et al., 2013) could increase the incidence of MetS. Inasmuch as nutrients and foods are not consumed separately and their simultaneous intake may have interaction, evaluation of dietary patterns can suggest a comprehensive view over the relationship between food and diseases. (Hu, 2002). Dietary patterns assess variety and combination of different foods in a diet and the frequency with which they are consumed (Castro‐Quezada et al., 2015). Two posterior (data‐driven) and prior (hypothesis‐oriented) methods have been used to identify dietary patterns (Babio et al., 2009). Posteriori methods derive patterns empirically based on the observed dietary intake using principal component analysis (PCA), cluster analysis, etc. (Fransen et al., 2014). Priori methods estimate the individuals’ adherence to dietary recommendations (Fransen et al., 2014; McNaughton, 2010). Dietary Approaches to Stop Hypertension (DASH) as one of the prior patterns is characterized by a high intake of fruits, vegetables, whole grains, and low‐fat dairy products, which was initially used to treat hypertension (Z Asemi et al., 2014; Zatollah Asemi et al., 2013). However, recent studies reported the protective effects of DASH diet on some diseases such as kidney, nonalcoholic fatty liver, and coronary heart diseases (Fung et al., 2008; Tyson et al., 2016; Xiao et al., 2020). The Mediterranean dietary pattern (MED) emphasizes the consumption of fruits, vegetables, legumes, whole grains, and types of food which is rich in monounsaturated fatty acids (MUFAs). Adherence to MED dietary pattern is associated with a lower risk of several chronic diseases such as diabetes and coronary heart disease and inflammatory and metabolic biomarkers (Fung et al., 2009; Gu et al., 2010; Martínez‐González et al., 2008; Rumawas et al., 2009).

Nowadays, studies are showing that MED dietary patterns may have protective effect on these diseases, due to low energy dense and low glycemic index (Asemi et al., 2014; Asemi et al., 2013). Limited data are available linking priori dietary patterns, including DASH and MED dietary patterns to Mets. For instance, several studies reported that the highest adherence to the DASH dietary pattern could reduce the risk of MetS (Pimenta et al., 2015; Saneei et al., 2015). However, some studies did not show any association between adherence to the dash diet and MetS and its components (Asghari et al., 2016; Folsom et al., 2007). Findings on adherence to MED dietary pattern and MetS are also inconsistent. Some studies conducted in different parts of the world revealed that adherence to the MED dietary pattern was associated with reduced of MetS incidence (Rumawas et al., 2009; Tortosa et al., 2007). However, some studies did not report a significant association between the MED dietary pattern and Mets (León et al., 2006; Mirmiran et al., 2015).

Most previous studies were conducted on western population and fewer studies have been done in non‐western nations, particularly in the Middle‐eastern populations, where the dietary patterns as well as prevalence of MetS are highly different from other parts of the world. So, the aim of this study was to evaluate the relationship between adherence to DASH and MED dietary patterns with the risk of MetS and its components in a large sample of Iranian adults.

## 2 MATERIAL AND METHODS

### 2.1 Study design and population

This cross‐sectional analysis was carried out on data derived from the recruitment phase of two prospective studies: Yazd Health Study (YaHS) and TAghzieh Mardom‐e‐Yazd (Yazd Nutrition Study) (TAMYZ). The investigated population included 10,000 residents of Yazd Greater Area who were within the age range of 20–69 years. The study design, sampling method, participants' characteristics, and data collection method were published elsewhere (Mirzaei et al., 2017).

Initially, laboratory and dietary data were collected from YaHS and TAMYZ studies, respectively. Later, the collected data from these two studies were merged. As a result, 2,221 participants entered this study after considering the exclusion criteria. Exclusion criteria included having 1) a history of cardiovascular diseases, strokes, diabetes, and cancers, 2) missing data, and 3) implausible energy intake (<600 kcal or >6,500 kcal).

### 2.2 Dietary assessment

The participants' dietary intakes were examined using the validated food frequency questionnaire (FFQ) containing 178 food items and 551 questions. The validity and reliability of this FFQ has been assessed in Iran (Mirmiran et al., 2010; Salehi‐Abargouei et al., 2020). Participants were asked to report their usual consumption frequency of food items in the last 12 months. Additionally, they were asked to report the type of milk, bread, and fat spread. The participants were provided with exact explanations about the portion sizes using household scales by the interviewer. In the next stage, single food items were combined into 40 groups based on their similarity and consumption rate (grams per day) and finally the nutrient intakes were computed (Mirmiran et al., 2010; Mirzaei et al., 2017; Salehi‐Abargouei et al., 2020).

### 2.3 Evaluation of anthropometric indices

Body weight was measured using a portable digital scale (Omron BF511 Inc. Nagoya, Japan) with an accuracy of 0.1 kg. All anthropometric measurements were calculated with three repetitions: before the interview begins and after completing one‐third and two‐thirds of the questionnaire. Height was measured in the standing position using a tape measure on a straight wall with an accuracy of 0.1 cm. The waist circumference (WC) was recorded to the nearest 0.5 cm using nonstretch tape placed midway between the iliac crest and lowest rib while participants were in the standing position. Hip circumference was also measured over the largest part of the buttocks with an accuracy of 0.5 cm. Body mass index (BMI) (kg/m^2^) was calculated using weight and height measurements according to the following formula: weight (kg)/height squared (m^2^).

### 2.4 Blood pressure measurement

Blood pressure was measured in the sitting position after the participants completed two‐thirds of the interview questions, so that the participants had already been in a resting position for at least 40 min. Blood pressure was measured three times with 5‐min intervals using Reichter electronic sphygmomanometers (Model N‐Champion, Riester GmbH, Germany) calibrated regularly. The second and third measurements' average was recorded as the participant's blood pressure.

### 2.5 Laboratory data

Fasting blood glucose (FBG) (mg/dl), high‐density lipoprotein cholesterol (HDL‐C), and triacylglycerol (TG) were measured based on the standard laboratory protocol using Pars Azmoon kits and calibrated Ciba Corning (Ciba Corp, Basle, Switzerland) auto‐analyzers.

### 2.6 Metabolic syndrome

According to the guidelines provided by the National Cholesterol Education Program Adult Treatment Panel III (Alberti et al., 2009), meeting three or more of the following criteria confirmed the presence of MetS: (1) WC >88 cm in women and >102 cm in men; (2) high levels of FBS (≥110 mg/dl); (3) low levels of HDL‐C < 50 mg/dl in women and HDL‐C < 40 mg/dl in men; (4) high levels of TG (≥150 mg/dl); and (5) increased blood pressure (systolic ≥130 mm Hg and diastolic ≥85 mm Hg).

### 2.7 Dietary patterns

Trichopoulou et al. (2005) method was used to calculate MED dietary scores. In this method, scores are assigned based on the eight food groups with a total maximum score of 8. One score was given to participants as long as they consumed an amount equal to or greater of the median intake for the daily serving of fruits, fish, vegetables, whole grains, legumes, nuts, and the ratio of grams of MUFA to saturated fatty acids (SFAs), whereas one score was given to the participants for the consumption of the lower amount of the median of meat (red meat, poultry, and processed meat) and dairy products. For example, we donated one point to participants who consumed vegetables greater than median intake. Finally, participants with high intake of fruits, fish, vegetables, whole grains, legumes, and nuts and low intake of meat and dairy products got a good score.

Fung et al. (2008) method was used to calculate the DASH dietary score. In this method, scores were also given based on 8 food groups. One score was given if the consumption of fruits, vegetables, nuts and legumes, low‐fat dairy products, and whole grains was within the highest quintile of participants. One score was also given if the daily intake of sodium, red and processed meats, and sweetened beverages was within the lowest quintile of participants. For instance, we donated one score to individuals placed on the top of the quintile of vegetable consumption. Individuals who have consumed high amount of fruits, vegetables, nuts and legumes, low‐fat dairy products, whole grains and low amount of sodium, red and processed meats, and sweetened beverages achieved a higher score.

Finally, the score of each participant for both dietary patterns was calculated by summing scores of eight food items. Then, participants were categorized into quintile based on their scores.

### 2.8 Other variables

Demographic and medical history questionnaires were also administered among the participants. Furthermore, the socioeconomic status, physical activity, and smoking status questionnaires were completed by the interviewer (Mirzaei et al., 2017).

### 2.9 Statistical analysis

The participants were classified based on the quintile of DASH and MED dietary patterns score. Higher scores indicated higher adherence and lower scores showed lower adherence. ANOVA and X^2^ tests were run to identify the differences across the DASH and MED quintiles for continuous and categorical variables, respectively. Multivariable logistic regression was conducted to investigate the association of adherence to DASH and MED dietary patterns with incidence of MetS. Moreover, our analysis was adjusted to the confounding variables such as age, education level, gender, physical activity, smoking, BMI, energy intake, and family history of diseases. All statistical analyses were performed in IBM SPSS version 22. The *p* < .05 was also considered statistically significant.

## 3 RESULTS

### 3.1 Study population characteristic

65.5% of the participants were under 50 years, and 34.5% were over the age of 50. The prevalence of MetS, abdominal obesity, low levels of HDL‐C, and high levels of TG, FBS, and blood pressure was 28.8%, 44.1%, 35%, 61.2%, 30.9%, and 69.5% respectively. Characteristics of the participants across quintiles of dietary patterns are presented in Table 1.

**TABLE 1:** Characteristics of participants according to the quintiles of dietary patterns

	Quintiles of DASH^a^				Quintiles of MED^a^			
	Q1	Q3	Q5	*p*‐ value^b^	Q1	Q3	Q5	*p*‐ value ^b^
Age (year)				.39				.35
20–40	46.9 (197)	41.8 (195)	38.7 (187)		43(142)	39.3(247)	40 (143)	
40–60	39 (164)	43 (204)	45 (217)		40 (132)	44 (277)	45 (160)	
≥60	13.6 (57)	14.2 (66)	15.1 (73)		16.4 (54)	16.3 (102)	13.8 (49)	
Sex	46.4(195)	48.3 (225)	46.5 (224)	.64	46.2 (152)	47.4 (297)	47.2 (167)	.79
Male	52.9(222)	51.3 (239)	52.7 (254)		53.8 (177)	51.8 (325)	52.3 (185)	
Female	26.95 ± 4.89	26.94 ± 4.87	27.18 ± 5.26		27.08 ± 4.82	26.98 ± 5.19	27.32 ± 5.25	
BMI (kg/m^2^)^c^	26.95 ± 4.89	26.94 ± 4.87	27.18 ± 5.26	.77	27.08 ± 4.82	26.98 ± 5.19	27.32 ± 5.25	.88
Waist circumference (cm)	93.68 ± 12.89	92.38 ± 12.5	93.36 ± 13.77	.63	93.93 ± 13.45	92.36 ± 13.09	93.72 ± 13.03	.23
Physical activity				.03				.13
Sedentary	24.3 (102)	26.6 (124)	33 (159)		23.7 (78)	33.2 (208)	28.2 (100)	
Moderate	37.1 (156)	36.9 (172)	34.9 (168)		37.1 (122)	34 (213)	33.1 (117)	
Active	34.3 (144)	33.3 (155)	28.2 (136)		35.9 (118)	29.2 (183)	33.3 (118)	
Education				.35				.14
Lower than high school diploma	48.5 (204)	52.7 (246)	52.6 (254)		52.8 (174)	55.5 (348)	52.8 (187)	
High school diploma or college	33.1 (139)	30 (140)	30.5 (147)		26.1 (86)	29 (182)	31.9 (113)	
University	18.1 (76)	16.5 (77)	15.6 (75)		20.1 (66)	14.4 (90 )	13.6 (48)	
Smoking								
Never and former	90 (378)	88.41 (412)	87.7 (423)	.46	90.27 (297)	90.27 (566)	87 (308 )	.02
Current	7.9 (33)	9 (42)	8.7 (42)		7.3 (24)	6.7 (42)	9.6 (34 )	
Ethnicity								
From Yazd	87.9 (369)	89.9 (419)	83.4 (402)	.07	87.8 (289)	87.1 (546)	82.5 (292)	.08
Not from Yazd	10.2 (43)	9.2 (43)	14.7 (71)		10 (33)	11.3 (71)	15.3 (54)	
Chronic disease history								
No	65.7 (276)	67.3 (3.14)	68.8 (332)	.96	69.9 (230)	66.3 (416)	66.3 (235)	.77
Yes	34.2 (144)	32.6 (152)	31 (150)		30 (99)	33.65 (211)	33.6 (119)	
Abdominal obesity	44 (185)	41.8 (195)	46.3 (223)	.41	45.9 (151)	39.9 (250)	44.1 (156)	.13
Elevated blood pressure	22.4 (94)	26.4 (123)	27.8 (134)	.44	25.2 (83 )	24.2 (152)	28.2 (100)	.24
High serum triacylglycerol	66.4 (279)	59.9 (279)	61.4 (296)	.13	63.2 (208)	61.8 (387)	59.6 (211)	.78
Low serum HDL‐C^d^	36.4 (153)	39.7 (185)	32.6 (157)	.10	34.3 (113)	31.7 (199)	33.6 (119)	.17
Abnormal glucose homeostasis	13.8 (58)	12.2 (57)	12.9 (62)	.63	15.5 (51)	12.1 (76)	12.7 (45)	.67
Average scores of dietary pattern	1.93 ± 1.07	3.02 ± 1.31	4.08 ± 1.05		1.69 ± 0.55	4	6.25 ± 0.47	

^a^ Data are reported as mean ±standard deviation (*SD*), otherwise explained,

^b^ ANOVA or Kruskal–Wallis test were used for continues variable and chi‐square test was used for categorical variable; statistical significance was set at the level of *p* ≤ .05.

^c^ BMI: body mass index,

^d^ HDL: high‐density lipoprotein cholesterol.

Participants in the highest quintile of DASH score had significantly lower levels of physical activity (*p* < .05) in comparison to those were in the first quintile. There was a significant difference in smoking status between participants in MED dietary pattern (*p* < .05). However, there was no significant association between other variables. So, smoking status was assessed separately in its own model in our analysis. In addition, individuals who smoke may not adhere properly to healthy dietary patterns.

### 3.2 Adherence of DASH and metabolic syndrome and its components

There was no significant relationship between adherence to DASH and the risk of MetS in the whole population (Table S1). However, the odds of MetS and abdominal obesity was lower in women who were in the highest quintiles of DASH in comparison to the first quintile after adjusting for confounding variables, respectively (OR: 0, 50 CI: 0.27–0.95, *p*‐ trend, .006) (OR: 0, 34 CI: 0.15–0.77, *p*‐ trend, .004). Furthermore, women in the third quintile of DASH had 34% lower odds for high TG levels after adjusting for age, energy intake, marital status, physical activity, education, smoking status, economic situation, ethnicity, and disease history (OR: 0.48, CI: 0.26–0.91). This significant relationship disappeared after further adjustment for BMI (OR: 0.56, CI: 0.31–1.03) (Tables 2 and 3).

**TABLE 2:** Multivariate adjusted OR and 95% CI for MetS based on dietary patterns’ quintile in women

	DASH ^a^				MED^a^			
	Q1	Q3	Q5	*p* for trend^b^	Q1	Q3	Q5	*p* for trend^b^
Metabolic syndrome^c^								
Crude	1	0.76 (0.52–1.13)	0.75 (0.51–1.10)	.24	1	0.76 (0.51–1.14)	0.88 (0.56–1.38)	.82
Model 1^d^	1	0.68 (0.45–1.04)	0.70 (0.47–1.06)	.16	1	0.68 (0.45–1.04)	0.84 (0.52–1.35)	.97
Model 2^e^	1	1.02 (0.69–1.50)	0.87 (0.59–1.27)	.84	1	0.86 (0.58–1.26)	1.03 (0.66–1.59)	.22
Model 3^f^	1	0.40 (0.21–0.74)	0.54 (0.29–0.86)	.008	1	0.81 (0.41–1.57)	0.94 (0.44–1.99)	.57
Model 4^g^	1	0.39 (0.20–0.74)	0.50(0.27–0.95)	.006	1	0.79 (0.40–1.56)	0.93 (0.43–1.99)	.58
Abdominal obesity								
Crude	1	0.86 (0.59–1.27)	0.88 (0.60–1.29)	.49	1	0.81 (0.55–1.19)	0.97 (0.63–1.49)	.78
Model 1^d^	1	0.71 (0.46–1.09)	0.79 (0.52–1.21)	.25	1	0.68 (0.44–1.04)	0.92 (0.56–1.49)	.98
Model 2^e^	1	0.91 (0.62–1.34)	0.88 (0.60–1.30)	.56	1	0.80 (0.54–1.18)	0.93 (0.60–1.45)	.94
Model 3^f^	1	0.56 (0.26–1.00)	0.44 (0.22–0.87)	.007	1	0.42 (0.20–0.87)	0.60 (0.26–1.35)	.45
Model 4^g^	1	0.58 (0.26–1.28)	0.34 (0.15–0.77)	.004	1	0.36 (0.15–0.86)	0.50 (0.19–1.32)	.32
Elevated blood pressure								
Crude	1	1.09 (0.71–1.68)	0.97 (0.62–1.53)	.36	1	1.07 (0.69–1.68)	1.15 (0.67–1.83)	.28
Model 1^d^	1	0.98 (0.61–1.58)	1.16 (0.73–1.85)	.45	1	1.00 (0.62–1.62)	1.17 (0.68–2.03)	.22
Model 2^e^	1	1.44 (0.97–2.14)	1.26 (0.85–1.85)	.19	1	1.11 (0.74–1.66)	1.13 (0.72–1.78)	.28
Model 3^f^	1	0.86 (0.43–1.70)	1.08 (0.54–2.17)	.63	1	0.87 (0.40–1.87)	0.97 (0.41–2.32)	.56
Model 4^g^	1	0.88 (0.44–1.76)	1.08 (0.54–2.18)	.60	1	0.91 (0.42–1.97)	1.01 (0.42–2.42)	.52
High serum triacylglycerol								
Crude	1	0.66 (0.44–0.98)	0.79 (0.53–1.18)	.99	1	0.72 (0.48–1.06)	0.82 (0.52–1.28)	.94
Model 1^d^	1	0.71 (0.47–1.08)	0.80 (0.53–1.21)	.82	1	0.73 (0.48–1.10)	0.77 (0.48–1.23)	.85
Model 2^e^	1	0.67 (0.44–1.00)	0.81 (0.54–1.22)	.87	1	0.72 (0.48–1.08)	0.86 (0.54–1.35)	.88
Model 3^f^	1	0.48 (0.26–0.91)	0.69 (0.36–1.32)	.79	1	0.92 (0.49–1.74)	1.51 (0.71–3.21)	.45
Model 4^g^	1	0.56 (0.31–1.03)	1.31 (0.67–2.54)	.79	1	0.87 (0.45–1.66)	1.51 (0.70–3.26)	.46
Low serum HDL‐C								
Crude	1	1.03 (0.71–1.49)	0.76 (0.53–1.09)	.12	1	0.94 (0.64–1.36)	0.97 (0.63–1.74)	.48
Model 1^d^	1	1.03 (0.71–1.50)	0.77 (0.53–1.12)	.13	1	0.93 (0.64–1.36)	0.96 (0.63–1.48)	.49
Model 2^e^	1	1.03 (0.71–1.50)	0.72 (0.49–1.04)	.07	1	0.92 (0.63–1.34)	0.88 (0.57–1.36)	.69
Model 3^f^	1	0.91 (0.52–1.60)	0.67 (0.38–1.19)	.19	1	1.43 (0.78–2.64)	0.94 (0.46–1.91)	.21
Model 4^g^	1	0.94 (0.54–1.66)	0.68 (0.38–1.21)	.21	1	1.42 (0.77–2.62)	0.94 (0.46–1.93)	.20
Abnormal glucose homeostasis								
Crude	1	0.79 (0.46–1.35)	0.88 (0.52–1.48)	.22	1	0.63 (0.38–1.05)	0.77 (0.44–1.35)	.58
Model 1^d^	1	0.70 (0.39–1.23)	0.83 (0.48–1.44)	.12	1	0.57 (0.33–0.97)	0.77 (0.42–1.40)	.62
Model 2^e^	1	1.04 (0.70–1.55)	0.95 (0.64–1.42)	.42	1	0.60 (0.40–0.90)	0.98 (0.63–1.51)	.78
Model 3^f^	1	0.58 (0.25–1.30)	0.72 (0.33–1.57)	.57	1	0.51 (0.23–1.14)	0.66 (0.26–1.65)	.36
Model 4^g^	1	0.63 (0.28–1.45)	0.74 (0.33–1.64)	.08	1	0.49 (0.22–1.11)	0.70 (0.27–1.77)	.44

^a^ Values are reported as odds ratio and 95% confidence interval.

^b^ Using the Mantel–Haenszel extension χ^2^ test. Statistical significance was set at the level of *p* ≤ .05.

^c^ Metabolic syndrome was defined as the presence of three or more of the following components: (1) abdominal adiposity (waist circumference >88 cm in women and >102 cm in men); (2) low serum HDL‐C < 50 mg/dl in women and HDL‐C < 40 mg/dl in men; (3) high serum triacylglycerol levels ≥150 mg/dl; (4) elevated blood pressure ≥130/85 mm Hg; (5) abnormal glucose homeostasis (fasting plasma glucose level ≥110 mg/dl).

^d^ Adjusted for age and energy intake (Kcal/day).

^e^ Adjusted for smoking status (never or former, current).

^f^ Adjusted for marriage status (married/single/divorce or widowed), physical activity (sedentary/moderate/active), education level (less than high school diploma/college/ university), job status (Not employed, employed), house status (home‐owner or tenant), number of family members (less than 4, more than five), house area (less than 100 meters, between 100 and 200 meters, more than 200 meters), ethnicity (from Yazd or not from Yazd), disease history (yes/ no) plus variables in models 1 and 2.

^g^ Additionally adjusted for BMI (kg/m^2^).

**TABLE 3:** Multivariate adjusted OR and 95% CI for MetS based on dietary patterns’ quintile in men

	DASH^a^				MED^a^			
	Q1	Q3	Q5	*p* for trend^b^	Q1	Q3	Q5	*p* for trend^b^
Metabolic syndrome^c^								
Crude	1	1.67 (0.90–3.08)	2.02 (0.98–3.69)	.07	1	0.80 (0.44–1.46)	1.20 (0.64–2.27)	.70
Model 1^d^	1	1.58 (0.84–2.97)	1.81 (0.98–3.35)	.14	1	0.84 (0.45–1.54)	1.32 (0.68–2.55)	.54
Model 2^e^	1	1.36 (0.89–2.07)	1.14 (0.74–1.75)	.59	1	0.88 (0.56–1.37)	1.04 (0.64–1.71)	.84
Model 3^f^	1	1.84 (0.71–4.74)	2.48 (0.99–6.18)	.08	1	0.93 (0.36–2.39)	1.37 (0.51–3.72)	.58
Model 4^g^	1	1.72 (0.63–4.69)	2.26 (0.87–5.89)	.23	1	1.07 (0.39–2.93)	1.57 (0.55–4.47)	.48
Abdominal obesity								
Crude	1	0.98 (0.61–1.59)	1.55 (0.99–2.45)	.14	1	0.71 (0.44–1.14)	0.93 (0.55–1.57)	.34
Model 1^d^	1	0.89 (0.54–1.45)	1.41 (0.89–2.24)	.27	1	0.72 (0.44–1.17)	0.99 (0.57–1.69)	.50
Model 2^e^	1	1.00 (0.61–1.61)	1.54 (0.97–2.44)	.13	1	0.68 (0.42–1.10)	0.92 (0.54–1.55)	.38
Model 3^f^	1	0.88 (0.42–1.88)	1.65 (0.81–3.53)	.08	1	0.74 (0.35–1.57)	0.91 (0.40–2.05)	.44
Model 4^g^	1	0.64 (0.24–1.70)	1.45 (0.59–3.58)	.35	1	1.28 (0.49–3.36)	0.89 (0.31–2.54)	.28
Elevated blood pressure								
Crude	1	1.43 (0.91–2.25)	1.59 (0.99–2.49)	.13	1	0.76 (0.49–1.19)	1.17 (0.72–1.89)	.23
Model 1^d^	1	1.41 (0.88–2.26)	1.45 (0.92–2.31)	.28	1	0.78 (0.49–1.23)	1.25 (0.75–2.08)	.17
Model 2^e^	1	1.09 (0.67–1.77)	0.89 (0.56–1.43)	.53	1	0.79 (0.47–1.34)	1.12 (0.61–2.05)	.91
Model 3^f^	1	1.31(0.65–2.65)	1.75 (0.89–3.44)	.06	1	0.74 (0.36–1.53)	1.45 (0.67–3.10)	.20
Model 4^g^	1	1.25 (0.61–2.55)	1.70 (0.86–3.38)	.10	1	0.84 (0.40–1.75)	1.53 (0.70–3.32)	.20
High serum triacylglycerol								
Crude	1	0.90 (0.61–1.32)	0.83 (0.56–1.22)	.30	1	1.26 (0.85–1.87)	0.90 (0.58–1.40)	.64
Model 14	1	0.84 (0.56–1.25)	0.82 (0.55–1.22)	.29	1	1.27 (0.85–1.89)	0.92 (0.59–1.45)	.77
Model 2^e^	1	0.91 (0.61–1.35)	0.84 (0.56–1.24)	.36	1	1.25 (0.83–1.86)	0.87 (0.56–1.36)	.63
Model 3^f^	1	0.92 (0.51–1.68)	0.84 (0.46–1.51)	.34	1	1.64 (0.87–3.07)	0.90 (0.46–1.79)	.33
Model 4^g^	1	1.33 (0.75–2.38)	1.12 (0.61–2.06)	.41	1	1.46 (0.77–2.77)	0.86 (0.43–1.72)	.29
Low serum HDL‐C								
Crude	1	1.36 (0.89–2.09)	0.96 (0.62–1.50)	.68	1	0.86 (0.54–1.35)	1.00 (0.60–1.66)	.74
Model 1^d^	1	1.36 (0.88–2.11)	1.01 (0.64–1.58)	.52	1	0.89 (0.56–1.41)	1.05 (0.63–1.76)	.98
Model 2^e^	1	1.41 (0.92–2.10)	0.97 (0.62–1.52)	.68	1	0.90 (0.56–1.42)	1.02 (0.61–1.69)	.86
Model 3^f^	1	1.58 (0.82–3.05)	0.94 (0.47–1.89)	.73	1	0.78 (0.36–1.67)	1.13 (0.50–2.54)	.83
Model 4^g^	1	1.64 (0.85–3.20)	0.97 (0.48–1.96)	.69	1	0.81 (0.38–1.75)	1.16 (0.51–2.62)	.30
Abnormal glucose homeostasis								
Crude	1	1.01 (0.56–1.81)	1.01 (0.56–1.82)	.98	1	0.93 (0.51–1.69)	0.84 (0.42–1.67)	.64
Model 1^d^	1	0.93 (0.50–1.72)	0.81 (0.44–1.50)	.52	1	0.94 (0.50–1.75)	0.84 (0.41–1.74)	.63
Model 2^e^	1	1.15 (0.75–1.77)	1.12 (0.73–1.72)	.62	1	0.99 (0.64–1.55)	1.09 (0.66–1.78)	.81
Model 3^f^	1	0.81 (0.31–2.09)	0.86 (0.34–2.15)	.66	1	1.57 (0.53–4.64)	1.35 (0.42–4.39)	.84
Model 4^g^	1	0.72 (0.27–1.89)	1.06 (0.99–1.13)	.51	1	1.65 (0.55–4.93)	1.40 (0.43–4.56)	.87

^a^ Values are reported as odds ratio and 95% confidence interval.

^b^ Using the Mantel–Haenszel extension χ^2^ test. Statistical significance was set at the level of *p* ≤ .05.

^c^ Metabolic syndrome was defined as the presence of three or more of the following components: (1) abdominal adiposity (waist circumference >88 cm in women and >102 cm in men); (2) low serum HDL‐C < 50 mg/dl in women and HDL‐C < 40 mg/dl in men; (3) high serum triacylglycerol levels ≥150 mg/dl; (4) elevated blood pressure ≥130/85 mm Hg; (5) abnormal glucose homeostasis (fasting plasma glucose level ≥110 mg/dl).

^d^ Adjusted for age and energy intake (Kcal/day).

^e^ Adjusted for smoking status (never or former, current).

^f^ Adjusted for marriage status (married/single/divorce or widowed), physical activity (sedentary/moderate/active), education level (less than high school diploma/college/ university), job status (Not employed, employed), house status (home‐owner or tenant), number of family members (less than 4, more than five), house area (less than 100 meters, between 100 to 200 meters, more than 200 meters), ethnicity (from Yazd or not from Yazd), disease history (yes/ no) plus variables in model 1 and 2.

^g^ Additionally adjusted for BMI (kg/m^2^).

### 3.3 Adherence of Mediterranean dietary pattern and metabolic syndrome and its components

There was no significant relationship between adherence to MED dietary pattern and the risk of MetS in the whole population (Table S1). Also, the same result was reported in the gender stratification model (Tables 2 and 3), whereas, women in the third quintile of MED dietary pattern in comparison to the first quintile had lower odds of high FBS levels after adjusting based on the model 1 (OR: 0, 57 CI: 0.33–0.97) and model 2 (OR: 0.60 CI: 0.40–0.90). In addition, moderate adherence to MED dietary pattern could decrease the odds of abdominal obesity in women after adjusting based on the model 4 (OR: 0, 36 CI: 0.15–0.86).

## 4 DISCUSSION

The present study revealed that greater adherence to the DASH dietary pattern was protectively associated with the risk of METs in the women. Moreover, adherence to DASH and MED dietary patterns decreased the odds of abdominal obesity in women and in the whole population. Moderate adherence to DASH and MED dietary patterns could decrease the odds of high TG levels and FBS levels in the women, respectively.

DASH and MED dietary patterns were considered at the same time in this study. Such studies can demonstrate a more comprehensive view of the association between diet quality and nutrition‐related diseases (Hu, 2002). To the best of our knowledge, this was the first study exploring the relationship between these two dietary patterns and MetS risk in a large sample of Iranian adults.

In confirmation of our results, a cross‐sectional study conducted among female nurses in Iran reported that women who were in the highest quartile of DASH had lower risk of MetS, abdominal obesity, and TG levels compared with those in the lowest quartile. However, no significant association was seen between adherence to DASH diet and abnormal FBS in this study (Saneei et al., 2015). Another study which prospectively assessed the association between DASH diet and risk of MetS reported such a similar result only in persons with low alcohol consumption in the Spanish adult population (Pimenta et al., 2015). In contrast with our findings, two studies reported that DASH dietary pattern had no significant association with the odds of abdominal obesity and high TG levels in Iranian and American adolescents (Liese et al., 2011; Saneei et al., 2013). Such discrepancies in the results can be attributed to the difference in participants’ age and gender. Women have a higher awareness and better knowledge of nutrition than men. They prefer eating healthier food such as fruits, vegetables, dairy products, and whole grain products to unhealthier food, whereas the consumption of unhealthier food like red meat and high GI foods is higher in men (Kiefer et al., 2005).

Several mechanisms may explain the health benefits of the DASH dietary pattern. In this dietary pattern, consumption of fruits and vegetables, high‐quality carbohydrates, and unsaturated vegetable oils was high, and intake of red and processed meat was low.

High consumption of dietary fiber attenuates indicators of metabolic abnormalities such as TG and abdominal obesity through delaying gastric emptying. Calcium, as another important ingredient of DASH, protects against the MetS through reduction of adiposity (Zemel et al., 2009). It is also essential to reduce fat absorption through formation of insoluble fatty acid soaps in the intestine. So, calcium plays an important role in reducing WC (Zemel et al., 2009).

Recent researches on the association between MED and MetS have also demonstrated contradictory results. A longitudinal study reported that adherence to MED dietary pattern was not significantly associated with the MetS risk and its component after 3 years of follow‐up in Iranian adult populations. In this study, the score of MED dietary pattern had been estimated using two methods. A cross‐sectional study revealed that adherence to MED dietary pattern was not related to MetS risk, but it was related to 70% lower risk of the blood pressure in Spanish adult populations (León et al., 2006; Mirmiran et al., 2015). On the contrary side, Tortosa et al. and Rumawas et al. showed the protective effects of MED dietary pattern on METs incidence in Spanish and American populations (Rumawas et al., 2009; Tortosa et al., 2007).

Although the consumption of fruits and vegetables, whole grains, and unsaturated vegetable oils were high in the MED dietary pattern, protective effects on MetS were not observed in the present study.

The difference in the study region may be one of the main reasons why such results were reported. The MED dietary pattern in the Mediterranean countries absolutely differs from other countries like Iran (Hoffman & Gerber, 2013). For example, the main part of cereals belongs to white rice and refined grains in Iran (Bahadoran et al., 2014). However, the majority of people consume brown rice and whole grains in the Mediterranean region (Hoffman & Gerber, 2013). In addition, there is a difference between the fish intake and the method of cooking which result in differences in the intake of omega‐3 between these two regions. For instance, olive oil is used to prepare fish in the Mediterranean countries. This oil may increase the nutritional value of fish through increased absorption of antioxidants, phenols, and vitamin (Hoffman & Gerber, 2013). At most times, in order to prepare fish and other foods, corn and sunflower oils, that contain less proportion of unsaturated fatty acid especially oleic acid than olive oil, were used in Iran (López Beceiro et al., 2011). Although olive oil is the main component in the diet in Mediterranean countries (Hoffman & Gerber, 2013), few people (more rich people) use this oil for cooking in Iran.

Differences in diagnostic criteria of MetS and the method used to obtain a MED dietary pattern score are other reasons for various results. Furthermore, higher prevalence of MetS in women than men in the present study as well as different hormone status, disparate sex psychosocial stressors, and lifestyle may justify these contradictory results between men and women (Tian et al., 2017). In addition, previous studies presented that old women were more vulnerable to have MetS compared with men. In other words, they claimed that the risk of having MetS increase, when women got older (Tian et al., 2017). But, in present study, there was no significant difference between men and women in terms of age.

Our study has also several strengths. First, this study included a large number of participants with a broad range collection of health‐related data covering both urban and rural areas. Second, a comprehensive and validated FFQ was applied for evaluating the dietary intake. Third, the confounding variables were adjusted as far as possible. However, the findings of the current study should be considered with several limitations. First, its cross‐sectional design made the researchers unable to extract the causal inferences. So, prospective studies are needed to confirm our findings. Second, although a validated FFQ was administered, some problems were existed such as 1) the same portion size was used for both sexes which may result in substantial errors in the estimation of nutrient intakes, 2) the United States Department of Agriculture's (USDA) food composition table (FCT) was used to calculate the energy and nutrient intakes for the majority of foods because of lack of existence of complete Iranian FCT. This point might not affect the correlation coefficients and the assessment of misclassifications, however, might lead to biased absolute intakes. Third, residual confounding from unknown or unmeasured factors could affect our findings. Finally, data on alcohol use were not collected due to local sensitivity.

## 5 CONCLUSION

This study reported that grater adherence to DASH dietary pattern could decrease the odds of METs and abdominal obesity in women. Although greater adherence to the MED dietary pattern did not affect the odds of MetS, moderate adherence to this dietary pattern decreased the odds of FBS levels and abdominal obesity in women. Further studies, especially more prospective cohort studies are required to clarify the exact causal relationship between these dietary patters and MetS.

## 6 ETHICAL REVIEW

The study's protocols and procedures were ethically reviewed and approved by a recognized ethical body (Ethics Committee of Shahid Sadoughi University of Medical Science with ethics code of (IR.SSU.SPH.REC.1397.08). This study does not involve any human or animal testing. Also, this study conforms to the Declaration of Helsinki, US, and/or European Medicines Agency Guidelines for human subjects.

## 7 INFORMED CONSENT

Written informed consent was obtained from all study participants.

## CONFLICTS OF INTEREST

All authors declared no personal or financial conflicts of interest.

## AUTHOR CONTRIBUTIONS

**Shirin Hassani Zadeh:** Conceptualization (equal); Data curation (equal); Formal analysis (equal); Funding acquisition (equal); Investigation (equal); Methodology (equal); Software (equal); Writing‐original draft (equal). **Amin Salehi‐Abargouei:** Conceptualization (equal); Formal analysis (equal); Methodology (equal); Software (equal); Visualization (equal); Writing‐review & editing (supporting). **Masoud Mirzaei:** Conceptualization (equal); Investigation (equal); Resources (equal); Validation (equal); Visualization (equal); Writing‐review & editing (equal). **Azadeh Nadjarzadeh:** Conceptualization (supporting); Visualization (supporting); Writing‐review & editing (supporting). **Mahdieh Hosseinzadeh:** Conceptualization (lead); Formal analysis (equal); Funding acquisition (equal); Investigation (equal); Methodology (equal); Project administration (equal); Supervision (lead); Validation (equal); Visualization (equal); Writing‐original draft (supporting); Writing‐review & editing (equal).

## Supporting information

Table S1

## References

[fsn32387-bib-0001] Alberti, K. , Eckel, R. H. , Grundy, S. M. , Zimmet, P. Z. , Cleeman, J. I. , Donato, K. A. , Fruchart, J.‐C. , James, W. P. T. , Loria, C. M. , & Smith, S. C. (2009). Harmonizing the metabolic syndrome: A joint interim statement of the international diabetes federation task force on epidemiology and prevention; national heart, lung, and blood institute; American heart association; world heart federation; international atherosclerosis society; and international association for the study of obesity. Circulation, 120(16), 1640–1645. 10.1161/CIRCULATIONAHA.109.192644 19805654

[fsn32387-bib-0002] Alswat, K. A. , Alnemari, A. K. , Alghamdi, I. , Almalki, A. A. , Al‐Thomali, B. , & Mahfouz, T. (2017). Prevalence of metabolic syndrome in the hospitalized psychiatric patients. Medical Archives (Sarajevo, Bosnia and Herzegovina), 71(6), 412–416. 10.5455/medarh.2017.71.412-416 PMC5788507 29416202

[fsn32387-bib-0003] Asemi, Z. , Samimi, M. , Tabassi, Z. , & Esmaillzadeh, A. (2014). The effect of DASH diet on pregnancy outcomes in gestational diabetes: A randomized controlled clinical trial. European Journal of Clinical Nutrition, 68(4), 490–495. 10.1038/ejcn.2013.296 24424076

[fsn32387-bib-0004] Asemi, Z. , Samimi, M. , Tabassi, Z. , Sabihi, S.‐S. , & Esmaillzadeh, A. (2013). A randomized controlled clinical trial investigating the effect of DASH diet on insulin resistance, inflammation, and oxidative stress in gestational diabetes. Nutrition, 29(4), 619–624. 10.1016/j.nut.2012.11.020 23466048

[fsn32387-bib-0005] Asghari, G. , Yuzbashian, E. , Mirmiran, P. , Hooshmand, F. , Najafi, R. , & Azizi, F. (2016). Dietary approaches to stop hypertension (DASH) dietary pattern is associated with reduced incidence of metabolic syndrome in children and adolescents. The Journal of Pediatrics, 174(178–184), e171. 10.1016/j.jpeds.2016.03.077 27156186

[fsn32387-bib-0006] Babio, N. , Bulló, M. , Basora, J. , Martínez‐González, M. A. , Fernández‐Ballart, J. , Márquez‐Sandoval, F. , Molina, C. , & Salas‐Salvadó, J. (2009). Adherence to the Mediterranean diet and risk of metabolic syndrome and its components. Nutrition, Metabolism and Cardiovascular Diseases, 19(8), 563–570. 10.1016/j.numecd.2008.10.007 19176282

[fsn32387-bib-0007] Bahadoran, Z. , Mirmiran, P. , Delshad, H. , & Azizi, F. (2014). White rice consumption is a risk factor for metabolic syndrome in tehrani adults: A prospective approach in Tehran lipid and glucose study. Archives of Iranian Medicine, 17, 435–440. 24916530

[fsn32387-bib-0008] Castanho, G. K. F. , Marsola, F. C. , Mclellan, K. C. P. , Nicola, M. , Moreto, F. , & Burini, R. C. (2013). Consumption of fruit and vegetables associated with the Metabolic Syndrome and its components in an adult population sample. Ciencia & Saude Coletiva, 18(2), 385–392. 10.1590/s1413-81232013000200010 23358764

[fsn32387-bib-0009] Castro‐Quezada, I. , Román‐Viñas, B. , & Serra‐Majem, L. (2015). The Mediterranean diet and nutritional adequacy: A review. Nutrients, 6(1):231–248. 10.3390/nu6010231 PMC3916858 24394536

[fsn32387-bib-0010] Fatahi, A. , Doosti‐Irani, A. , & Cheraghi, Z. (2020). Prevalence and incidence of metabolic syndrome in Iran: A systematic review and meta‐analysis. International Journal of Preventive Medicine, 11, 64. 10.4103/ijpvm.IJPVM_489_18 32577194 PMC7297433

[fsn32387-bib-0011] Fitzmorris, P. S. , Colantonio, L. D. , Perez, E. T. , Smith, I. , Kakati, D. D. , & Malik, T. A. (2015). Impact of metabolic syndrome on the hospitalization rate of Crohn's disease patients seen at a tertiary care center: A retrospective cohort study. Digestion, 91(3), 257–262. 10.1159/000380763 25833139

[fsn32387-bib-0012] Folsom, A. R. , Parker, E. D. , & Harnack, L. J. (2007). Degree of concordance with DASH diet guidelines and incidence of hypertension and fatal cardiovascular disease. American Journal of Hypertension, 20(3), 225–232. 10.1016/j.amjhyper.2006.09.003 17324731 PMC2020811

[fsn32387-bib-0013] Fransen, H. P. , May, A. M. , Stricker, M. D. , Boer, J. M. A. , Hennig, C. , Rosseel, Y. , & Beulens, J. W. J. (2014). A posteriori dietary patterns: How many patterns to retain? The Journal of Nutrition, 144(8), 1274–1282. 10.3945/jn.113.188680 24872222

[fsn32387-bib-0014] Fung, T. T. , Chiuve, S. E. , McCullough, M. L. , Rexrode, K. M. , Logroscino, G. , & Hu, F. B. (2008). Adherence to a DASH‐style diet and risk of coronary heart disease and stroke in women. Archives of Internal Medicine, 168(7), 713–720. 10.1001/archinte.168.7.713 18413553

[fsn32387-bib-0015] Fung, T. T. , Rexrode, K. M. , Mantzoros, C. S. , Manson, J. E. , Willett, W. C. , & Hu, F. B. (2009). Mediterranean diet and incidence and mortality of coronary heart disease and stroke in women. Circulation, 119(8), 1093. 10.1161/CIRCULATIONAHA.108.816736 19221219 PMC2724471

[fsn32387-bib-0016] Gu, Y. , Luchsinger, J. A. , Stern, Y. , & Scarmeas, N. (2010). Mediterranean diet, inflammatory and metabolic biomarkers, and risk of Alzheimer's disease. Journal of Alzheimer's Disease, 22(2), 483–492. 10.3233/JAD-2010-100897 PMC3022949 20847399

[fsn32387-bib-0017] Hoffman, R. , & Gerber, M. (2013). Evaluating and adapting the Mediterranean diet for non‐Mediterranean populations: A critical appraisal. Nutrition Reviews, 71(9), 573–584. 10.1111/nure.12040 24032362

[fsn32387-bib-0018] Hu, F. B. (2002). Dietary pattern analysis: A new direction in nutritional epidemiology. Current Opinion in Lipidology, 13(1), 3–9. 10.1097/00041433-200202000-00002 11790957

[fsn32387-bib-0019] Kiefer, I. , Rathmanner, T. , & Kunze, M. (2005). Eating and dieting differences in men and women. Journal of Men's Health and Gender, 2(2), 194–201. 10.1016/j.jmhg.2005.04.010

[fsn32387-bib-0020] Klemsdal, T. O. , Holme, I. , Nerland, H. , Pedersen, T. R. , & Tonstad, S. (2010). Effects of a low glycemic load diet versus a low‐fat diet in subjects with and without the metabolic syndrome. Nutrition, Metabolism and Cardiovascular Diseases, 20(3), 195–201. 10.1016/j.numecd.2009.03.010 19502017

[fsn32387-bib-0021] LaHaye, S. A. , Hollett, P. M. , Vyselaar, J. R. , Shalchi, M. , Lahey, K. A. , & Day, A. G. (2005). Comparison between a low glycemic load diet and a Canada Food Guide diet in cardiac rehabilitation patients in Ontario. The Canadian Journal of Cardiology, 21(6), 489–494. 15917877

[fsn32387-bib-0022] Lakka, H.‐M. , Laaksonen, D. E. , Lakka, T. A. , Niskanen, L. K. , Kumpusalo, E. , Tuomilehto, J. , & Salonen, J. T. (2002). The metabolic syndrome and total and cardiovascular disease mortality in middle‐aged men. JAMA, 288(21), 2709–2716. 10.1001/jama.288.21.2709 12460094

[fsn32387-bib-0023] Lazo, M. , Hernaez, R. , Eberhardt, M. S. , Bonekamp, S. , Kamel, I. , Guallar, E. , Koteish, A. , Brancati, F. L. , & Clark, J. M. (2013). Prevalence of nonalcoholic fatty liver disease in the United States: The Third National Health and Nutrition Examination Survey, 1988–1994. American Journal of Epidemiology, 178(1), 38–45. 10.1093/aje/kws448 23703888 PMC3698993

[fsn32387-bib-0024] León, E. A. , Henriquez, P. , & Serra‐Majem, L. (2006). Mediterranean diet and metabolic syndrome: A cross‐sectional study in the Canary Islands. Public Health Nutrition, 9(8A), 1089–1098. 10.1017/S1368980007668487 17378946

[fsn32387-bib-0025] Liese, A. D. , Bortsov, A. , Günther, A. L. B. , Dabelea, D. , Reynolds, K. , Standiford, D. A. , Liu, L. , Williams, D. E. , Mayer‐Davis, E. J. , D'Agostino, R. B. , Bell, R. , & Marcovina, S. (2011). Association of DASH diet with cardiovascular risk factors in youth with diabetes mellitus: The SEARCH for Diabetes in Youth study. Circulation, 123(13), 1410–1417. 10.1161/circulationaha.110.955922 21422385 PMC4669039

[fsn32387-bib-0026] Linardakis, M. , Bertsias, G. , Sarri, K. , Papadaki, A. , & Kafatos, A. (2008). Metabolic syndrome in children and adolescents in Crete, Greece, and association with diet quality and physical fitness. Journal of Public Health, 16(6), 421–428. 10.1007/s10389-008-0191-z

[fsn32387-bib-0027] López Beceiro, J. , Artiaga, R. , Gracia‐Fernández, C. , Tarrío‐Saavedra, J. , Naya, S. , & Mier, J. (2011). Comparison of olive, corn, soybean and sunflower oils by PDSC. Journal of Thermal Analysis and Calorimetry, 104, 169–175. 10.1007/s10973-010-1165-2

[fsn32387-bib-0028] Lutsey, P. L. , Steffen, L. M. , & Stevens, J. (2008). Dietary intake and the development of the metabolic syndrome: The Atherosclerosis Risk in Communities study. Circulation, 117(6), 754–761. 10.1161/CIRCULATIONAHA.107.716159 18212291

[fsn32387-bib-0029] Martínez‐González, M. Á. , De la Fuente‐Arrillaga, C. , Nunez‐Cordoba, J. , Basterra‐Gortari, F. , Beunza, J. J. , Vazquez, Z. , & Bes‐Rastrollo, M. (2008). Adherence to Mediterranean diet and risk of developing diabetes: Prospective cohort study. BMJ, 336(7657), 1348–1351. 10.1136/bmj.39561.501007.BE 18511765 PMC2427084

[fsn32387-bib-0030] McNaughton, S. A. (2010). Dietary patterns and diet quality: Approaches to assessing complex exposures in nutrition. Australasian Epidemiologist, 17(1), 35.

[fsn32387-bib-0031] Mirmiran, P. , Esfahani, F. H. , Mehrabi, Y. , Hedayati, M. , & Azizi, F. (2010). Reliability and relative validity of an FFQ for nutrients in the Tehran lipid and glucose study. Public Health Nutrition, 13(5), 654–662. 10.1017/S1368980009991698 19807937

[fsn32387-bib-0032] Mirmiran, P. , Moslehi, N. , Mahmoudof, H. , Sadeghi, M. , & Azizi, F. (2015). A longitudinal study of adherence to the Mediterranean dietary pattern and metabolic syndrome in a non‐Mediterranean population. International Journal of Endocrinology and Metabolism, 13(3), 10.5812/ijem.26128v2 PMC4584365 26425127

[fsn32387-bib-0033] Mirzaei, M. , Salehi‐Abargouei, A. , Mirzaei, M. , & Mohsenpour, M. A. (2017). Cohort profile: The Yazd Health Study (YaHS): A population‐based study of adults aged 20–70 years (study design and baseline population data). International Journal of Epidemiology, 47(3), 697–698h. 10.1093/ije/dyx231 29186588

[fsn32387-bib-0034] O'Neill, S. , & O'Driscoll, L. (2015). Metabolic syndrome: A closer look at the growing epidemic and its associated pathologies. Obesity Reviews, 16(1), 1–12. 10.1111/obr.12229 25407540

[fsn32387-bib-0035] Pimenta, A. M. , Toledo, E. , Rodriguez‐Diez, M. C. , Gea, A. , Lopez‐Iracheta, R. , Shivappa, N. , Hébert, J. R. , & Martinez‐Gonzalez, M. A. (2015). Dietary indexes, food patterns and incidence of metabolic syndrome in a Mediterranean cohort: The SUN project. Clinical Nutrition, 34(3), 508–514. 10.1016/j.clnu.2014.06.002 24975512 PMC4870043

[fsn32387-bib-0036] Reaven, G. M. (2005). The insulin resistance syndrome: Definition and dietary approaches to treatment. Annual Review of Nutrition, 25, 391–406. 10.1146/annurev.nutr.24.012003.132155 16011472

[fsn32387-bib-0037] Rumawas, M. E. , Meigs, J. B. , Dwyer, J. T. , McKeown, N. M. , & Jacques, P. F. (2009). Mediterranean‐style dietary pattern, reduced risk of metabolic syndrome traits, and incidence in the Framingham Offspring Cohort. The American Journal of Clinical Nutrition, 90(6), 1608–1614. 10.3945/ajcn.2009.27908 19828705 PMC3152203

[fsn32387-bib-0038] Salari, N. , Doulatyari, P. K. , Daneshkhah, A. , Vaisi‐Raygani, A. , Jalali, R. , Jamshidi, P. K. , Abdi, A. , Mohammadi, M. , & Kazeminia, M. (2020). The prevalence of metabolic syndrome in cardiovascular patients in Iran: A systematic review and meta‐analysis. Diabetology and Metabolic Syndrome, 12(1), 96. 10.1186/s13098-020-00605-4 33292427 PMC7607701

[fsn32387-bib-0039] Salehi‐Abargouei, A. , Zimorovat, A. , Moghtaderi, F. , Amiri, M. , Raeisi‐Dehkordi, H. , Mohyadini, M. , & Mirzaei, M. (2020). Validity and reproducibility of a semi‐quantitative multiple‐choice food frequency questionnaire in adults living in central Iran. Research Square, 10.21203/rs.3.rs-15529/v1

[fsn32387-bib-0040] Saneei, P. , Fallahi, E. , Barak, F. , Ghasemifard, N. , Keshteli, A. H. , Yazdannik, A. R. , & Esmaillzadeh, A. (2015). Adherence to the DASH diet and prevalence of the metabolic syndrome among Iranian women. European Journal of Nutrition, 54(3), 421–428. 10.1007/s00394-014-0723-y 24906470

[fsn32387-bib-0041] Saneei, P. , Hashemipour, M. , Kelishadi, R. , Rajaei, S. , & Esmaillzadeh, A. (2013). Effects of recommendations to follow the Dietary Approaches to Stop Hypertension (DASH) diet v. usual dietary advice on childhood metabolic syndrome: A randomised cross‐over clinical trial. British Journal of Nutrition, 110(12), 2250–2259. 10.1017/s0007114513001724 23773316

[fsn32387-bib-0042] Tian, X. , Xu, X. , Zhang, K. , & Wang, H. (2017). Gender difference of metabolic syndrome and its association with dietary diversity at different ages. Oncotarget, 8(43), 73568. 10.18632/oncotarget.20625 29088727 PMC5650282

[fsn32387-bib-0043] Tortosa, A. , Bes‐Rastrollo, M. , Sanchez‐Villegas, A. , Basterra‐Gortari, F. J. , Nuñez‐Cordoba, J. M. , & Martinez‐Gonzalez, M. A. (2007). Mediterranean diet inversely associated with the incidence of metabolic syndrome. Diabetes Care, 30(11), 2957–2959. 10.2337/dc07-1231 17712023

[fsn32387-bib-0044] Trichopoulou, A. , Bamia, C. , & Trichopoulos, D. (2005). Mediterranean diet and survival among patients with coronary heart disease in Greece. Archives of Internal Medicine, 165(8), 929–935. 10.1001/archinte.165.8.929 15851646

[fsn32387-bib-0045] Tyson, C. C. , Lin, P.‐H. , Corsino, L. , Batch, B. C. , Allen, J. , Sapp, S. , Barnhart, H. , Nwankwo, C. , Burroughs, J. , & Svetkey, L. P. (2016). Short‐term effects of the DASH diet in adults with moderate chronic kidney disease: A pilot feeding study. Clinical Kidney Journal, 9(4), 592–598. 10.1093/ckj/sfw046 27478603 PMC4957723

[fsn32387-bib-0046] Xiao, M.‐L. , Lin, J.‐S. , Li, Y.‐H. , Liu, M. , Deng, Y.‐Y. , Wang, C.‐Y. , & Chen, Y.‐M. (2020). Adherence to the Dietary Approaches to Stop Hypertension (DASH) diet is associated with lower presence of non‐alcoholic fatty liver disease in middle‐aged and elderly adults. Public Health Nutrition, 23(4), 674–682. 10.1017/S1368980019002568 31566148 PMC10200450

[fsn32387-bib-0047] Zemel, M. B. , Teegarden, D. , Van Loan, M. , Schoeller, D. A. , Matkovic, V. , Lyle, R. M. , & Craig, B. A. (2009). Dairy‐rich diets augment fat loss on an energy‐restricted diet: A multicenter trial. Nutrients, 1(1), 83–100. 10.3390/nu1010083 22253969 PMC3257590

