# Supplementary material for: The association between dietary approaches to stop hypertension diet and mediterranean diet with metabolic syndrome in a large sample of Iranian adults: YaHS and TAMYZ Studies
Source: Food Sci Nutr. 2021 Jun 11;9(7):3932–41. doi: 10.1002/fsn3.2387 (PMC8269560; doi:10.1002/fsn3.2387)
Supplement: Supplementary file 1 — Table S1 [file FSN3-9-3932-s001.docx]

|  | DASH ^1^ | | | | MED^1^ | | | |
| --- | --- | --- | --- | --- | --- | --- | --- | --- |
|  | Q1 | Q3 | Q5 | P for trend ^2^ | Q1 | Q3 | Q5 | P for trend^2^ |
| Metabolic syndrome ^3^ |  |  |  |  |  |  |  |  |
| Crude | 1 | (1.29 - 0.69) 0.95 | (1.38 - 0.74) 1.07 | 0.95 | 1 | (1.06 - 0.55) 0.76 | (1.39 - 0.68) 0.97 | 0.85 |
| Model 1^4^ | 1 | (1.25 - 0.63) 0.89 | (1.32 - 0.68) 0.95 | 0.75 | 1 | (1.03 - 0.52) 0.73 | (1.44 - 0.67) 0.98 | 0.71 |
| Model 2 ^5^ | 1 | 1.15 (0.87 - 1.51) | 0.98 (0.75 - 1.29) | 0.92 | 1 | 0.86 (0.65 - 1.14) | 1.03 (0.75 - 1.41) | 0.61 |
| Model 3^6^ | 1 | (1.13 - 0.42) 0.69 | (1.40 - 0.54) 0.87 | 0.67 | 1 | (1.41 - 0.49) 0.83 | (1.93 - 0.60) 1.08 | 0.46 |
| Model 4^7^ | 1 | (1.09 - 0.40) 0.66 | (1.30 - 0.50) 0.81 | 0.49 | 1 | (1.45 - 0.48) 0.84 | (1.98 - 0.60) 1.09 | 0.43 |
| Abdominal obesity |  |  |  |  |  |  |  |  |
| Crude | 1 | 0.90 (0.68 - 1.17) | (1.43 - 0.84) 1.10 | 0.91 | 1 | (1.02 - 0.59) 0.78 | (1.28 - 0.69) 0.94 | 0.47 |
| Model 1^4^ | 1 | (1.11 - 0.59) 0.81 | (1.37 - 0.74) 1.01 | 0.99 | 1 | (0.98 - 0.53) 0.72 | (1.37 - 0.67) 0.96 | 0.65 |
| Model 2 ^5^ | 1 | 0.94 (0.71 - 1.23 ) | 1.10 (0.84 - 1.45) | 0.77 | 1 | 0.77 (0.58 - 1.01) | 0.93 (0.68 - 1.27) | 0.40 |
| Model 3^6^ | 1 | (1.14 - 0.44) 0.71 | (1.32 - 0.51) 0.82 | 0.33 | 1 | (0.90 - 0.33) 0.55 | (1.24 - 0.41) 0.72 | 0.93 |
| Model 4^7^ | 1 | (1.20 - 0.37) 0.67 | (1.28 - 0.39) 0.71 | 0.13 | 1 | (1.09 - 0.32) 0.59 | (1.26 - 0.33) 0.64 | 0.16 |
| Elevated blood pressure |  |  |  |  |  |  |  |  |
| Crude | 1 | (1.68 - 0.90) 1.23 | (1.82 - 0.98) 1.34 | 0.09 | 1 | (1.25 - 0.67) 0.91 | (1.62 - 0.81) 1.14 | 0.1 |
| Model 1^4^ | 1 | (1.65 - 0.85) 1.18 | (1.76 - 0.92) 1.27 | 0.22 | 1 | (1.23 - 0.63) 0.88 | (1.76 - 0.84) 1.21 | 0.07 |
| Model 2 ^5^ | 1 | 1.27 (0.94 - 1.71) | 1.09 (0.81 - 1.46) | 0.49 | 1 | 0.99 (0.72 - 1.36) | 1.13 (0.79 - 1.62) | 0.28 |
| Model 3^6^ | 1 | (1.83 - 0.70) 1.14 | (2.25 - 0.88) 1.41 | 0.06 | 1 | (1.35 - 0.48) 0.81 | (2.08 - 0.68) 1.19 | 0.21 |
| Model 4^7^ | 1 | (1.86 - 0.71) 1.15 | (2.26 - 0.88) 1.41 | 0.07 | 1 | (1.45 - 0.51) 0.86 | (2.19 - 0.71) 1.25 | 0.19 |
| High serum triacylglycerol |  |  |  |  |  |  |  |  |
| Crude | 1 | (0.99 - 0.57) 0.75 | 0.80 (0.61 - 1.05) | 0.37 | 1 | (1.26 - 0.71) 0.93 | (1.16 - 0.63) 0.85 | 0.68 |
| Model 1^4^ | 1 | (1.02 - 0.58) 0.77 | (1.09 - 0.62) 0.82 | 0.58 | 1 | (1.27 - 0.72) 0.95 | (1.17 - 0.61) 0.85 | 0.76 |
| Model 2 ^5^ | 1 | 0.76 (0.57 - 1.00) | 0.82 (0.62 - 1.08) | 0.49 | 1 | 0.93 (0.71 - 1.24) | 0.86 (0.63 - 1.18) | 0.71 |
| Model 3^6^ | 1 | (1.06 - 0.46) 0.70 | (1.15 - 0.49) 0.75 | 0.53 | 1 | (1.80 - 0.75) 1.16 | (1.84 - 0.69) 1.12 | 0.89 |
| Model 4^7^ | 1 | (1.36 - 0.60) 0.91 | (1.88 - 0.78) 1.21 | 0.58 | 1 | (1.68 - 0.68) 1.07 | (1.80 - 0.66) 1.09 | 0.82 |
| Low serum HDL-C |  |  |  |  |  |  |  |  |
| Crude | 1 | (1.50 - 0.87) 1.14 | (1.12 - 0.64) 0.84 | 0.30 | 1 | (1.19 - 0.67) 0.89 | (1.34 - 0.71) 0.97 | 0.88 |
| Model 1^4^ | 1 | (1.55 - 0.88) 1.17 | (1.13 - 0.64) 0.85 | 0.43 | 1 | (1.23 - 0.69) 0.92 | ) 1.000.72 - 1.39) | 0.61 |
| Model 2 ^5^ | 1 | 1.17 (0.89 - 1.54) | 0.82 (0.62 - 1.08) | 0.21 | 1 | 0.90 (0.68 - 1.21) | 0.94 (0.68 - 1.30) | 0.99 |
| Model 3^6^ | 1 | (1.84 - 0.80) 1.22 | (1.21 - 0.51) 0.78 | 0.50 | 1 | (1.88 - 0.73) 1.18 | (1.76 - 0.61) 1.03 | 0.25 |
| Model 4^7^ | 1 | (1.92 - 0.83) 1.26 | (1.24 - 0.52) 0.80 | 0.56 | 1 | (1.91 - 0.74) 1.19 | (1.79 - 0.62) 1.05 | 0.22 |
| Abnormal glucose homeostasis |  |  |  |  |  |  |  |  |
| Crude | 1 | (1.28 - 0.58) 0.87 | (1.35 - 0.62) 0.92 | 0.30 | 1 | (1.10 - 0.51) 0.75 | (1.22 - 0.51) 0.79 | 0.45 |
| Model 1^4^ | 1 | (1.21 - 0.52) 0.79 | (1.24 - 0.55) 0.82 | 0.12 | 1 | (1.05 - 0.47) 0.70 | (1.26 - 0.50) 0.80 | 0.50 |
| Model 2 ^5^ | 1 | 1.09 (0.81 - 1.45) | 1.04 (0.78 - 1.39) | 0.86 | 1 | 0.77 (0.57 - 1.03) | 1.02 (0.74 - 1.42) | 0.97 |
| Model 3^6^ | 1 | (1.20 - 0.36) 0.66 | (1.34 - 0.42) 0.75 | 0.06 | 1 | (1.43 - 0.42) 0.77 | (1.66 - 0.41) 0.83 | 0.54 |
| Model 4^7^ | 1 | (1.23 - 0.36) 0.66 | (0.42 - 1.36) 0.76 | 0.06 | 1 | (0.42 - 1.46) 0.79 | (1.77 - 0.44) 0.88 | 0.62 |

^1^ Values are reported as odds ratio and 95 % confidence interval.

**Supplementary table-** Multivariate adjusted OR and 95% CI for MetS based on dietary patterns’ quintile in whole population.

^2^ Using the Mantel–Haenszel extensionχ2test.Statistical significance was set at the level of P≤0.05.

^3^ Metabolic syndrome was defined as the presence of three or more of the following components: (1) abdominal adiposity (waist circumference > 88 cm in women and >102 cm in men); (2) low serum HDL-C <50 mg/dl in women and HDL-C <40 mg/dl in men; (3) high serum triacylglycerol levels ⩾150 mg/dl; (4) elevated blood pressure ⩾130/85 mm Hg; (5) abnormal glucose homeostasis (fasting plasma glucose level⩾110 mg/dl).

^4^ Adjusted for age,gender, and energy intake (Kcal/day)

^5^ Adjusted for smoking status (never or former, current)

^6^ Adjusted for marriage status (married/single/divorce or widowed), physical activity (sedentary/moderate/active), education level (less than high school diploma/college/ university), job status (Not employed, employed), house status (home-owner or tenant), number of family members (less than 4, more than five), house area (less than 100 meters, between 100 to 200 meters, more than 200 meters), ethnicity (from Yazd or not from Yazd), disease history (yes/ no) plus variables in model 1 and 2.

^7^ Additionally adjusted for BMI (kg/m^2^)
